# Laboratory Evaluation of Interferences Associated with Factor XIa Inhibitors Asundexian and Milvexian in Routine Coagulation Assays

**DOI:** 10.3390/diagnostics14171991

**Published:** 2024-09-09

**Authors:** Gavin T. Buckley, Maeve P. Crowley, James V. Harte

**Affiliations:** 1 Department of Haematology, Cork University Hospital, Wilton, T12 DC4A Cork, Ireland; 2 EOLAS Research Group, Cork University Hospital, Wilton, T12 DC4A Cork, Ireland

**Keywords:** activated partial thromboplastin time, anticoagulants, asundexian, direct oral anticoagulants, coagulation, factor XIa, milvexian, prothrombin time, thrombosis

## Abstract

Direct oral anticoagulants (DOACs) are increasingly used for the treatment of thrombosis. While inhibitors of factor IIa and factor Xa have shown effectiveness, the risk of bleeding remains a significant concern. Recently, direct factor XIa inhibitors—including asundexian and milvexian—have emerged as potential anticoagulation therapies, based on clinical observations that patients with factor XIa deficiencies seldom present with spontaneous bleeding tendencies. The interferences associated with DOACs in routine and specialised coagulation assays are well-described; however, the interferences associated with emerging FXIa inhibitors are largely uncharacterised. Here, we briefly report the impact of asundexian and milvexian in routine coagulation assays using in vitro plasma-based systems. Asundexian and milvexian induce concentration-dependent prolongations in APTT-based assays with curvilinear regressions, which may be suitable for the measurement of pharmacodynamic effects at peak levels ex vivo. We also report differential sensitivities of APTT-based assays—particularly at higher FXIa inhibitor concentrations—highlighting the clinical need for an extensive evaluation of interferences associated with FXIa inhibitors in coagulation assays.

## 1. Introduction

Anticoagulation therapy is a mainstay in the prevention and treatment of arterial and venous thrombosis. Since the introduction of direct oral anticoagulants (DOACs), direct inhibitors of factor IIa (FIIa) and factor Xa (FXa) have been increasingly favoured over vitamin K antagonists (VKAs) due to superior pharmacokinetic and pharmacodynamic profiles [1,2]. However, the risk of concomitant bleeding remains a significant concern with the administration of DOACs: the annual rate of DOAC-associated major bleeding ranges from 2.1% to 4.9% [3,4,5], whilst the lifetime risk of major bleeding remains unquantified due to a lack of long-term DOAC safety data [6]. DOAC-associated bleeding is also associated with an increased risk of DOAC discontinuation, contributing to greater risk of all-cause mortality, stroke and systemic embolism, and myocardial infarction [7].

Alternative anticoagulation therapies that minimise the risk of bleeding without compromising antithrombotic efficacy are, therefore, highly desirable. Recently, inhibition of factor XI (FXI) and activated factor XI (FXIa) have emerged as potentially safer approaches to anticoagulation due on the differential role of FXI/XIa in physiological haemostasis and pathological thrombosis.

Haemostasis in vivo is initiated by the binding of factor VII (FVII) or VIIa (FVIIa) to tissue factor exposed at the site of vascular injury [8]. The resulting FVIIa:tissue factor complex converts inactive factor X (FX) to activated FX (FXa), which then converts finite quantities of prothrombin into thrombin and initiates fibrin deposition [8].

In addition to activating FX, the FVIIa: tissue factor complex converts inactive factor IX (FIX) to activated FIX (FIXa), which sustains thrombin generation post-vascular injury through the amplification of thrombin generation. The importance of the intrinsic FVIIIa–FIXa pathway in haemostasis is reflected by the severe bleeding disorders associated with inherited FIX or FVIII deficiencies [9].

Although FXI is also activated during haemostasis, thrombosis formation is largely independent of FXIa [10]. FXI/XIa is restricted to the consolidation of thrombi and the determination of resistance to fibrinolysis through activation of FIX [10]; indeed, patients with haemophilia C or congenital FXI deficiency infrequently present with bleeding tendencies [11], and even patients with severe deficiencies rarely exhibit spontaneous bleeding [12].

However, the capacity of the FVIIa:tissue factor complex to propagate pathological thrombi may be limited beyond the initial site of vascular injury. It has been proposed that intraluminal progression may be dependent on the consolidatory activity of FXI/XIa [10]. Interestingly, in patients with haemophilia C or congenital FXI deficiency, reduced FXI/FXIa is associated with lower rates of venous thromboembolic events [13] whereas such events are substantially increased in patients with higher levels of FXI [14].

As such, it is expected that the inhibition of FXI/XIa will contribute less to the risk of major bleeding than contemporary direct FIIa and FXa inhibitors. Several approaches to target FXI/XIa are under clinical investigation, including antisense oligonucleotides and monoclonal antibodies, which function by lowering FXI concentrations to replicate congenital FXI deficiency, and synthetic small molecules, which function by inhibiting the procoagulant activity of FXI/XIa.

**Figure 1:**
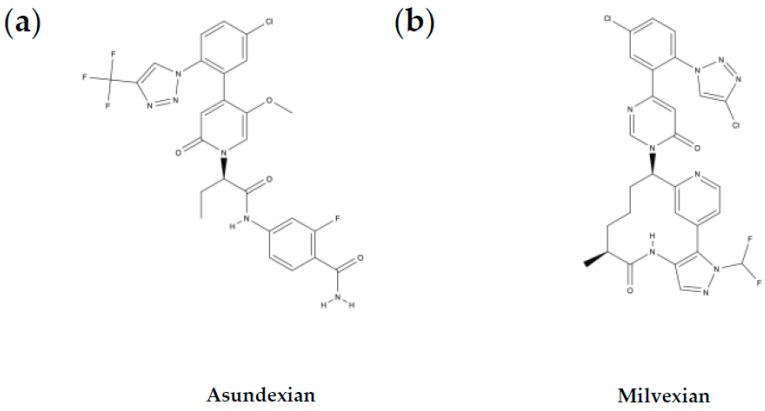
Chemical structures of investigational FXIa inhibitors asundexian (**a**) and milvexian (**b**). Structure visualized with MolView (Available at: https://molview.org/ [Accessed: 22 August 2024]).

Two small molecule inhibitors of FXIa (Figure 1) are currently in clinical trial following supportive evidence in a series of phase 2 trials [15,16,17,18,19]: asundexian is under investigation in the phase 3 OCEANIC-STROKE trial (NCT05686070); and, milvexian in the phase 3 LIBREXIA-ACS (NCT05754957), LIBREXIA-AF (NCT05757869), and LIBREXIA-STROKE (NCT05702034) trials.

Overall, the OCEANIC and LIBREXIA programs aim to advance anticoagulation therapy by uncoupling the management of pathological thrombosis from the bleeding risk inadvertently associated with current FIIa and FXa inhibitors.

Direct FIIa and FXa inhibitors are well-known to be associated with extensive interference in routine and specialised coagulation assays [4]. Although, factor IIa and factor Xa inhibitors do not require continuous biological monitoring, it is recognised by clinicians and laboratorians that the assessment of direct FIIa and FXa inhibitor concentration and/or activity may be required in some circumstances [20,21,22]. It is likely that FXIa inhibitors—including asundexian and milvexian—will require similar laboratory support; however, the effect of direct FXIa inhibitors on routine coagulation assays is not well-defined in the literature at present. Therefore, in this study, we preliminarily evaluated the potential for asundexian- and milvexian-associated interferences in routine coagulation assays using in vitro plasma-based systems.

## 2. Materials and Methods

### 2.1. Preparation of Human-Derived Plasma

Human-derived plasma samples were obtained as commercial lyophilised preparations of Control and Calibrator Material from Siemens Healthineers (Erlangen, Germany) and HYPHEN BioMed (Neuville-sur-Oise, France). Plasma samples were reconstituted with distilled water at ambient temperature, according to the manufacturer’s recommendations.

Additional details regarding the human-derived plasma samples herein for testing are available in Appendix A (Table A1).

### 2.2. Preparation of Anticoagulant Solutions

Asundexian (catalogue number: HY-137431) and milvexian (catalogue number: HY- 125856) were obtained from MedChemExpress (Sollentuna, Sweden). Stock solutions were prepared by dissolving each anticoagulant in dimethyl sulfoxide (DMSO), as per the manufacturer’s recommendations, to a concentration of 1 mg/mL; stock solutions were then either diluted directly in the reconstituted human-derived plasma or diluted in phosphate-buffered saline (PBS; pH 7.4) to obtain intermediate concentrations of 100 µg/mL, 10 µg/mL, or 1 µg/mL, before final dilution in the reconstituted human-derived plasma prior to analysis (0–4000 ng/mL).

### 2.3. Routine and Specialised Coagulation Assays

The prothrombin time (PT), the activated partial thromboplastin time (APTT), and fibrinogen assay according to von Clauss were performed using Dade^®^ Innovin^®^ or Thromborel^®^ S, Dade^®^ Actin^®^ FS or Dade^®^ Actin^®^ FSL, and Dade^®^ Thrombin reagents, respectively. Immunoturbidometric measurements of fibrin-derived D-dimer were performed using INNOVANCE^®^ D-Dimer. Factor XIa activity was measured by a one-stage clotting assay using a commercial immunoadsorbed plasma with a coagulation factor XIa activity of ≤1%.

The reference ranges used were: a PT of 9.5–11.1 s and INR of 0.86–1.12 for Dade^®^ Innovin^®^; a PT of 9.6–13.0 s and INR of 0.88–1.21 for Thromborel^®^ S; an APTT of 21.0–29.0 s and an APTT ratio of 0.84–1.16 for Dade^®^ Actin^®^ FS; an APTT of 25.0–32.0 s and an APTT ratio of 1.00–1.28 for Dade^®^ Actin^®^ FSL; a fibrinogen concentration of 1.70–4.10 g/L for Dade^®^ Thrombin; and a D-dimer concentration of <0.5 mg/L.

The PT, APTT, fibrinogen, and fibrin-derived D-dimer were performed on a CN-6000 analyser (Sysmex, Kobe, Japan) and one-stage clotting assays were performed on a CS-2500 analyser (Sysmex, Kobe, Japan), which were monitored and maintained according to the manufacturer’s recommendations. To minimise variation, a single lot of reagents for each routine coagulation assay was used throughout this study.

Human-derived plasma samples were initially assayed after reconstitution. Then, asundexian or milvexian was added, and samples were assayed after incubation with direct FXIa inhibitors for 5 min at ambient temperature with gentle agitation.

Additional details regarding the human-derived plasma samples herein for testing are available in Appendix A (Table A2).

### 2.4. Statistical Analysis

All datapoints were generated from separate aliquots of human-derived plasma and separate experiments. The Shapiro–Wilk test was used to determine whether the data were parametric: continuous variables with normal distributions were expressed as means and standard deviations. Statistical differences were evaluated by the one-way ANOVA test with a post hoc Dunnett test for multiple comparisons. A *p*-value of less than 0.05 was considered statistically significant (*, *p* < 0.05; **, *p* < 0.01; ***, *p* < 0.001).

The extent of interference associated with direct FXIa inhibitors was defined as the final concentration of anticoagulant required to cause a twofold prolongation in the clotting time (2 × CT), as previous described [23,24,25,26].

Data were collected using Microsoft Excel (version 2018; Microsoft Corporation, Redmond, WA, USA). Statistical analyses were performed using GraphPad Prism (version 10.1.1; GraphPad Software, Boston, MA, USA).

## 3. Results

To evaluate asundexian- and milvexian-associated interferences in routine coagulation assays, aliquots of standard human plasma (Table A1) were spiked in vitro with direct FXIa inhibitors and assayed for the prothrombin time (PT), the activated partial thromboplastin time (APTT), fibrinogen, and fibrin-derived D-dimer. The selected concentrations are representative of maximum plasma concentrations reported [27,28].

### 3.1. Prothrombin Time (PT)

Two Quick-based PT assays were evaluated for sensitivity to asundexian and milvexian, with different thromboplastin sources: Dade^®^ Innovin^®^ contains recombinant human thromboplastin, whereas Thromborel^®^ S contains human placenta-derived thromboplastin.

**Figure 2:**
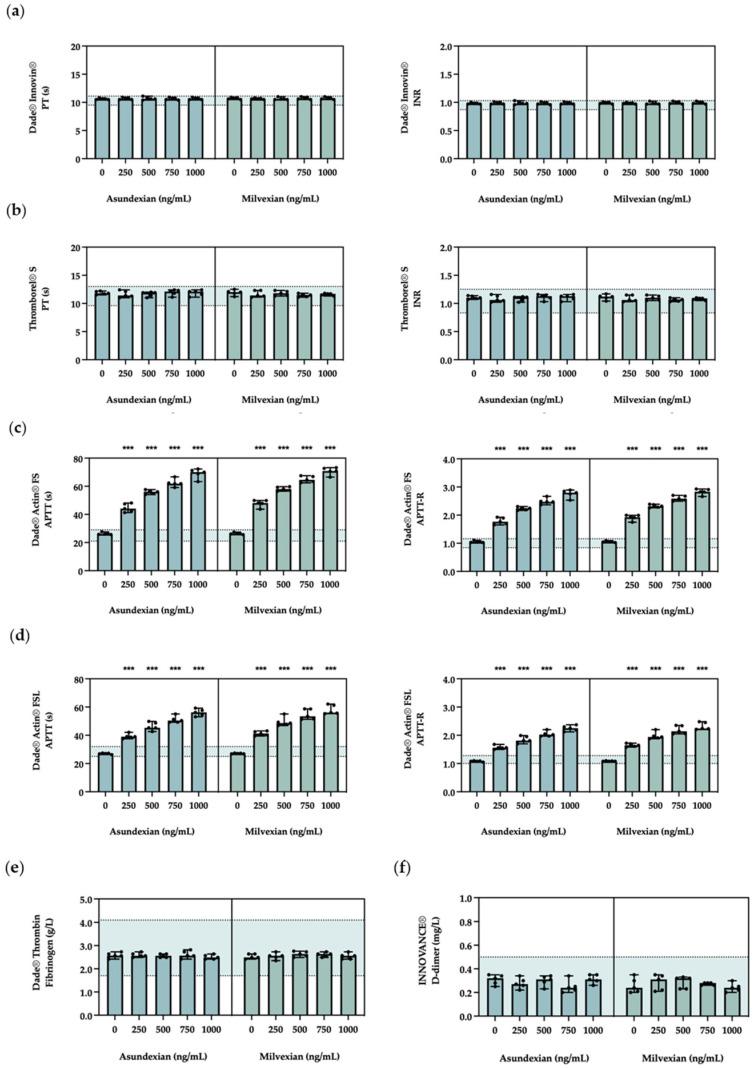
Interference associated with FXIa inhibitors in routine coagulation assays. The impact of select concentrations of asundexian and milvexian (0–1000 ng/mL) on Dade^®^ Innovin^®^ PT (**a**), Thromborel S^®^ PT (**b**), Dade^®^ Actin^®^ FS APTT (**c**), Dade^®^ Actin^®^ FSL APTT (**d**), Dade^®^ Thrombin (**e**), and INNOVANCE^®^ D-dimer (**f**) assays were determined in human-derived plasma. Data are presented as the mean (bars) with standard deviation, using a scatter dot plot. For each assay, five independent replicates of plasma spiked with asundexian or milvexian, respectively, were assayed (*n* = 5). Horizontal dashed lines represent normal ranges (Tables S1 and S2). Abbreviations: APTT, activated partial thromboplastin time; APTT-R, APTT ratio; DDi, D-dimer; FIB, fibrinogen; INR, international normalised ratio; PT, prothrombin time. Probability value (one-way ANOVA with post hoc Dunnett test): ***, *p* < 0.001.

The Quick-based clotting times and associated international normalised ratios (INRs) of human-derived plasma were insensitive to increasing concentrations of asundexian and milvexian, irrespective of the thromboplastin source (Figure 2a,b; extended details in Tables S1 and S2). Control and Calibrator Material with clotting times in clinically relevant ranges were also insensitive to increasing concentrations of asundexian and milvexian (Tables S3 and S4).

### 3.2. Activated Partial Thromboplastin Time (APTT)

Two ellagic acid-based APTT assays were evaluated for sensitivity to asundexian and milvexian, with different phospholipid compositions: Dade^®^ Actin^®^ FS contains purified soy phosphatides, whereas Dade^®^ Actin^®^ FSL contains a mixture of soy and rabbit brain phosphatides.

In contrast to the Quick-based PT assays, the ellagic acid-based clotting times and associated APTT ratios (APTT-Rs) were sensitive to increasing concentrations of asundexian and milvexian (Figure 2c,d; extended details in Tables S1 and S2). Control and Calibrator Material with clotting times in clinically relevant ranges were also sensitive to increasing concentrations of asundexian and milvexian, and it was noted that the anticoagulant effect was greater when there was an existing prolongation of ellagic acid-based clotting times (Tables S3 and S4).

Furthermore, differential sensitivities—particularly at higher anticoagulant concentrations—were observed for the two ellagic acid-based APTT assays: at an asundexian concentration of 1000 ng/mL, the clotting time with Dade^®^ Actin^®^ FS was 69.1 ± 3.5 s versus 56.1 ± 2.0 s with Dade^®^ Actin^®^ FSL (*p* < 0.001; extended details in Table S1); at a milvexian concentration of 1000 ng/mL, the ellagic acid-based clotting time with Dade^®^ Actin^®^ FS was 70.8 ± 2.6 s versus 58.1 ± 2.8 s with Dade^®^ Actin^®^ FSL (*p* < 0.001; extended details in Table S2).

### 3.3. Fibrinogen and D-Dimer

Concentrations of fibrinogen and fibrin-derived D-dimer were insensitive to increasing concentrations of asundexian and milvexian (Figure 2e,f; extended details in Tables S1 and S2). Control and Calibrator Material with fibrinogen and fibrin-derived D-dimer concentrations in clinically relevant ranges were also insensitive to increasing concentrations of asundexian and milvexian (Tables S3 and S4).

### 3.4. Activated Partial Thromboplastin Time (APTT) Testing May Be Useful for the Measurement of Plasma Levels of Asundexian and Milvexian

**Figure 3:**
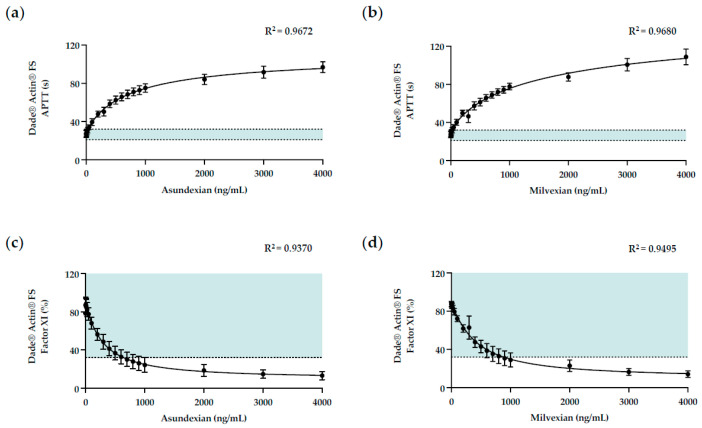
Interference associated with FXIa inhibitors in APTT-based assays. The impact of increasing concentrations of asundexian and milvexian (0–4000 ng/mL) on APTT clotting time (**a**,**b**) and FXI activity (**c**,**d**) assayed with Dade^®^ Actin^®^ FS were determined in human-derived plasma. Data are presented as the mean (closed circles) with standard deviation, using an XY plot. For each assay, five independent replicates of plasma spiked with asundexian or milvexian, respectively, were assayed (N = 5; *n* = 5). Horizontal dashed lines represent normal ranges (Tables S5 and S6). Abbreviations: APTT, activated partial thromboplastin time.

To further evaluate asundexian and milvexian interferences in APTT-based assays and to determine whether the APTT may be predictive for FXIa inhibitors ex vivo, a broader range of asundexian and milvexian concentrations—from 0 ng/mL to 4000 ng/mL—were spiked into five human-derived plasmas (Table A1) and assayed for APTT clotting times and FXIa concentrations with Dade^®^ Actin^®^ FS (Figure 3).

Asundexian and milvexian induced concentration-dependent prolongations in APTT clotting times with curvilinear regressions (Figure 3a,b): the clotting times were within the normal reference range when the concentrations of asundexian and milvexian were ≤50 ng/mL and were prolonged steadily thereafter (Tables S3 and S4).

At the higher concentrations of asundexian and milvexian tested, the extent of interference plateaued (Figure 3a,b): at an asundexian concentration of 4000 ng/mL, the APTT clotting time with Dade^®^ Actin^®^ FS was prolonged to 96.9 ± 5.6 s (extended details in Table S5), with a relative 3.6-fold change compared to the baseline; at a milvexian concentration of 4000 ng/mL, the clotting time with Dade^®^ Actin^®^ FS was prolonged to 108.8 ± 8.2 s (extended details in Table S6), with a relative 4-fold change compared to the baseline.

It was noted that asundexian-associated interferences in APTT clotting times were less potent than milvexian-associated interferences, supported by the respective 2 × CT concentrations for Dade^®^ Actin^®^ FS: the 2 × CT concentration for asundexian was 427.8 ± 54 ng/mL versus 356.7 ± 32 ng/mL for milvexian.

Asundexian and milvexian concomitantly induced concentration-dependent reductions in FXIa activities with curvilinear regressions (Figure 3c,d): the factor activities were within the normal reference range when the concentrations of asundexian and milvexian were ≤200 ng/mL and were reduced steadily thereafter (Tables S3 and S4).

At the highest concentration of asundexian and milvexian tested, the extent of interference in the one-stage clotting assay was moderate to severe (Figure 3c,d): at an asundexian concentration of 4000 ng/mL, the factor XI concentration with Dade^®^ Actin^®^ FS was apparently reduced to 14.2% ± 3.4 (extended details in Table S5) at a relative −6.6-fold compared to the baseline; at a milvexian concentration of 4000 ng/mL, the factor XI concentration with Dade^®^ Actin^®^ FS was apparently reduced to 13.2% ± 4.3 (extended details in Table S6) at a relative difference of −6.6-fold compared to the baseline.

It was also noted that a multi-dilutional analysis of human-derived plasma led to an increasing reduction in the effect of asundexian and milvexian in the one-stage clotting assay, consistent with the presence of a non-specific ‘inhibitor’ effect (Table S7).

## 4. Discussion

Direct FIIa inhibitors and direct FXa inhibitors are associated with extensive interference in coagulation assays requiring laboratory support to avoid the misinterpretation of results [29]. Therefore, as FXIa inhibitors—including asundexian and milvexian—emerge as possible antithrombotic therapies with potentially minimised bleeding risks, there is a clinical need to understand the impact of direct FXIa inhibitors in laboratory investigations.

Here, we evaluated asundexian- and milvexian-associated interferences in routine coagulation assays by spiking human-derived plasma with FXIa inhibitors in vitro and assaying for the PT, the APTT, fibrinogen, and fibrin-derived D-dimer—assays that may be commonly encountered by clinicians and laboratorians if FXIa inhibitors emerge from the OCEANIC and LIBREXIA programs.

Due to the underlying principle of the reagents involved, only the FXIa-dependent assays were shown to be sensitive to asundexian- and milvexian-associated interferences in vitro: no interferences were observed for the PT, fibrinogen, nor fibrin-derived D-dimer assays, whereas a curvilinear relationship of interference was observed between APTT-based clotting times and increasing asundexian and milvexian concentrations, respectively, due to apparent reductions in FXI concentrations [30,31].

As asundexian- and milvexian-associated interferences are limited to FXIa-dependent assays, the proportion of assays sensitive to direct FXIa inhibitors will likely be less than those sensitive to direct FIIa inhibitors and direct FXa inhibitors [29]. The restriction of these interferences to FXIa-dependent assays may therefore pose a reduced challenge to clinical laboratories, which are increasingly accustomed to handling the presence of DOACs in patient-derived plasma samples.

Similar to the risk associated with direct FIIa inhibitors and direct FXa inhibitors, if uncommunicated or unrecognised, the presence of asundexian or milvexian in patient-derived plasma samples may lead to an apparent moderate-to-severe intrinsic factor deficiency, consistent with the presence of a non-specific ‘inhibitor’ by multi-dilutional one-stage clotting assays. Asundexian and milvexian concentrations exceeding 200 ng/mL were associated with apparent FXI deficiencies in this study, which were in line with both the pharmacokinetic properties of asundexian and milvexian [32,33] and the apparent FXI activities observed in the presence of ascending doses of milvexian by Perera et al. [34].

As such, clinicians and laboratorians must be aware that FXIa inhibitors may become additional pre-analytical reasons for prolonged APTT-based clotting times in isolation, particularly as concentrations of FXIa inhibitors increase, and communicate appropriately.

Nevertheless, as a rapid and routinely available coagulation assay, APTT-based clotting times may therefore be useful in the measurement of FXIa inhibitors ex vivo. However, the relationships between asundexian and milvexian concentrations and APTT-based clotting times were noted to be curvilinear, reaching a plateau at higher concentrations, which may limit the clinical utility of the APTT in supratherapeutic situations.

As previously noted for direct FIIa and FXa inhibitors [35,36,37,38,39], different assays may have differing sensitivities to the presence of direct FXIa inhibitors. Dade^®^ Actin^®^ FS was shown to have a higher sensitivity when compared to Dade^®^ Actin^®^ FSL, particularly at higher concentrations of asundexian and milvexian. The differences are likely due to the distinct phospholipid compositions of Dade^®^ Actin^®^ FS and Dade^®^ Actin^®^ FSL, respectively: while both reagents contain 0.1 mM ellagic acid as an activator, the phospholipid composition in Dade^®^ Actin^®^ FS differs from other reagents, with a higher ratio of unsaturated-to-saturated fatty acids and an absence of phosphatidylserine [40]. The differences in phospholipid composition have been reported to enhance the sensitivity of Dade^®^ Actin^®^ FS to factor XI deficiency [41]. Therefore, although the APTT may be useful for the rapid assessment of asundexian and milvexian concentrations in vitro, the sensitivity of any given APTT-based reagent must be carefully considered by both clinicians and laboratorians.

Currently, however, there are no published therapeutic ranges for asundexian nor milvexian due to the investigational status of these FXIa inhibitors in the OCEANIC and LIBREXIA programs. Therefore, although the concentrations of asundexian and milvexian evaluated herein span the maximum plasma concentrations reported [27,28], no definitive conclusions can be made regarding the correlation of haemorrhagic or thrombotic complications with FXIa inhibitor concentrations determined using an APTT-based assay in clinical situations where asundexian or milvexian require laboratory monitoring.

Furthermore, based on the respective 2 × CT concentrations for asundexian and milvexian, a normal APTT-based clotting time may not be suitable for the exclusion of FXIa inhibitors ex vivo, depending on the sensitivity of the reagent, the coagulation factor activities in the sample, and the dose of asundexian or milvexian administered [27].

Overall, a more extensive evaluation of coagulation assays and their respective sensitivities to asundexian- and milvexian-associated interferences in patient-derived plasma samples is required before the suitability of APTT-based assays for the measurement of FXIa inhibitors can be considered.

Our study described herein is primarily limited by the testing of a small number of commercially available human-derived plasma samples rather than patient-derived samples. Although commercial Control and Calibrator Materials are derived from human plasma, we cannot exclude interferences introduced during manufacture. Moreover, the addition of FXIa inhibitors to human-derived plasma may not reflect patient-derived samples due to variability in patient factor activities or the effect of drug metabolites [42].

## 5. Conclusions

In conclusion, this in vitro study shows that asundexian and milvexian induce significant prolongations in routine FXIa-dependent assays. Routine APTT testing may be suitable for the measurement of the pharmacodynamic effects of asundexian and milvexian at peak levels ex vivo; however, clinicians and laboratorians should be aware that a normal APTT may not exclude the presence of either asundexian or milvexian. The responsivity—and feasibility—of individual APTT assays to increasing concentrations of FXIa inhibitors requires further investigation in patient-derived samples.

## Data Availability

All data generated for this manuscript are included herein. Raw data are available from the corresponding author upon reasonable request.

## Supplementary Materials

The following supporting information can be downloaded at https://www.mdpi.com/article/10.3390/diagnostics14171991/s1, Table S1: Absolute mean values (standard deviation) of routine coagulation assay parameters in human plasma at select concentrations of asundexian; Table S2: Absolute mean values (standard deviation) of routine coagulation assay parameters in human plasma at select concentrations of milvexian; Table S3: Absolute mean values (standard deviation) of routine coagulation assay parameters in Control and Calibrator Material at select concentrations of asundexian; Table S4: Absolute mean values (standard deviation) of routine coagulation assay parameters in Control and Calibrator Material at select concentrations of milvexian; Table S5: Absolute mean values (standard deviation) of APTT-based assay parameters in human plasmas at different concentrations of asundexian; Table S6: Absolute mean values (standard deviation) of APTT-based assay parameters in human plasmas at different concentrations of milvexian; Table S7. Multi-dilutional analysis of factor XI activity in human plasmas at 1000 ng/mL of asundexian and milvexian.

## Appendix A

**Table A1:** Control and Calibrator Material—additional details.

Material	Assays Performed	Catalogue No.	Lot No.	Source
Standard Human Plasma	PT, APTT, Fibrinogen, D-dimer, FXI-OS	10446238 ORKL17	503299	Siemens Healthineers
Ci-Trol^®^ 1	APTT, FXI-OS	10445601 291070	564878	Siemens Healthineers
Ci-Trol^®^ 2	PT, APTT	10445602 291071	548542	Siemens Healthineers
Ci-Trol^®^ 3	PT, APTT	10445603 291072	556574	Siemens Healthineers
Control Plasma N	APTT, FXI-OS	10446234 ORKE41	507922	Siemens Healthineers
Control Plasma P	Fibrinogen	10446471 OUPZ17	556748	Siemens Healthineers
INNOVANCE^®^ Control 2	D-dimer	10446005 OPDY03	575513	Siemens Healthineers
BIOPHEN ™ Plasma Calibrator	APTT, FXI-OS	22101	FB06641A	HYPHEN BioMed
BIOPHEN ™ Normal Control Plasma	APTT, FXI-OS	223201	FB06661C	HYPHEN BioMed

**Table A2:** Routine and specialised coagulation testing—additional details.

Assay	Reagent	Catalogue No.	Lot No.	Source
PT	Dade^®^ Innovin^®^ Thromborel^®^ S	10445704 B4212 10484202 OUHP49	564635 568155	Siemens Healthineers
APTT	Dade^®^ Actin^®^ FS Dade^®^ Actin^®^ FSL Calcium chloride	10445710 B4218 10445714 B421902 10446232 ORHO37	562452 562755 563935	Siemens Healthineers
Fibrinogen	Dade^®^ Thrombin Dade^®^ Owren’s Buffer	10445721 B4233 10445724 B4234	567438 569953	Siemens Healthineers
D-Dimer	INNOVANCE^®^ D-Dimer	10445980 OPBP07	01214	Siemens Healthineers
Factor XIa	Factor XI Deficient Plasma	10446316 OSDF13	503360	Siemens Healthineers
