# Supplementary material for: Laboratory Evaluation of Interferences Associated with Factor XIa Inhibitors Asundexian and Milvexian in Routine Coagulation Assays
Source: Diagnostics (Basel). 2024 Sep 9;14(17):1991. doi: 10.3390/diagnostics14171991 (PMC11394090; doi:10.3390/diagnostics14171991)
Supplement: Supplementary file 1 [file diagnostics-14-01991-s001.zip › diagnostics-3174255-supplementary.pdf]

diagnostics

*Article*

# Laboratory Evaluation of Interferences Associated with Factor XIa Inhibitors Asundexian and Milvexian in Routine Coagulation Assays

# Supplementary Material

**Gavin T. Buckley [1,2], Maeve P. Crowley [1,2] and James V. Harte [1,2]***

[1] Department of Haematology, Cork University Hospital, Wilton, Cork, Ireland, T12 DC4A
2 EOLAS Research Group, Cork University Hospital, Wilton, Cork, Ireland, T12 DC4A
* Correspondence: jharte@ucc.ie

**Table S1.** Absolute mean values (standard deviation) of routine coagulation assay parameters in human plasma at select concentrations of asundexian

| | | | **Asundexian** | | | | |
|---|---|---|---|---|---|---|---|
| **Assay** | **Parameter** | **Reference Range** | **0 ng/mL** | **250 ng/mL** | **500 ng/mL** | **750 ng/mL** | **1000 ng/mL** |
| **Dade® Innovin®** | PT (s) | 9.5 – 11.1 | 10.6 (0.1) | 10.7 (0.2) | 10.7 (0.3) | 10.6 (0.2) | 10.6 (0.2) |
| | INR | 0.86 – 1.12 | 0.98 (0.01) | 0.99 (0.02) | 0.99 (0.03) | 0.98 (0.02) | 0.98 (0.02) |
| **Thromborel® S** | PT (s) | 9.6 – 13.0 | 11.9 (0.3) | 11.6 (0.6) | 11.6 (0.4) | 11.9 (0.5) | 11.9 (0.5) |
| | INR | 0.88 – 1.21 | 1.11 (0.03) | 1.08 (0.01) | 1.08 (0.02) | 1.11 (0.02) | 1.11 (0.05) |
| **Dade® Actin® FS** | APTT (s) | 21.0 – 29.0 | 26.3 (0.8) | **44.6 (3.0)** | **55.8 (1.4)** | **62.3 (2.7)** | **69.1 (3.5)** |
| | APTT-R | 0.84 – 1.16 | 1.05 (0.03) | **1.79 (0.12)** | **2.23 (0.05)** | **2.49 (0.11)** | **2.77 (0.14)** |
| **Dade® Actin® FSL** | APTT (s) | 25.0 – 32.0 | 27.0 (0.5) | **39.3 (1.6)** | **46.3 (2.5)** | **51.1 (1.0)** | **56.1 (2.0)** |
| | APTT-R | 1.00 – 1.28 | 1.08 (0.02) | **1.57 (0.07)** | **1.85 (0.12)** | **2.04 (0.10)** | **2.24 (0.10)** |
| **Dade® Thrombin** | FIB (g/L) | 1.70 – 4.10 | 2.58 (0.11) | 2.60 (0.09) | 2.57 (0.12) | 2.62 (0.12) | 2.51 (0.10) |
| **INNOVANCE® D-dimer** | DDi (mg/L) | < 0.5 | 0.31 (0.04) | 0.28 (0.05) | 0.30 (0.04) | 0.25 (0.05) | 0.31 (0.04) |

Data is presented as mean ± standard deviation. Data in bold indicate statistically significant differences (one-way ANOVA with post hoc Dunnett testing: $p < 0.001$) compared to samples without asundexian (0 ng/mL). Abbreviations: APTT, activated partial thromboplastin time; APTT-R, activated partial thromboplastin time ratio; DDi, D-dimer; FIB, fibrinogen; INR, international normalised ratio; PT, prothrombin time. For each assay, five independent replicates of reconstituted standard human-derived plasma were spiked with asundexian or milvexian, respectively, were assayed ($n = 5$).

**Table S2.** Absolute mean values (standard deviation) of routine coagulation assay parameters in human plasma at select concentrations of milvexian

| | | | **Milvexian** | | | | |
|---|---|---|---|---|---|---|---|
| **Assay** | **Parameter** | **Reference Range** | **0 ng/mL** | **250 ng/mL** | **500 ng/mL** | **750 ng/mL** | **1000 ng/mL** |
| **Dade® Innovin®** | PT (s) | 9.5 – 11.1 | 10.7 (0.1) | 10.6 (0.2) | 10.6 (0.2) | 10.7 (0.2) | 10.7 (0.2) |
| | INR | 0.96 – 1.12 | 0.99 (0.02) | 0.98 (0.01) | 0.98 (0.02) | 0.99 (0.02) | 0.99 (0.02) |
| **Thromborel® S** | PT (s) | 9.6 – 13.0 | 12.1 (0.4) | 11.1 (0.5) | 11.8 (0.5) | 11.6 (0.3) | 11.7 (0.2) |
| | INR | 0.88 – 1.21 | 1.11 (0.05) | 1.09 (0.06) | 1.1 (0.04) | 1.06 (0.03) | 1.08 (0.02) |
| **Dade® Actin® FS** | APTT (s) | 21.0 – 29.0 | 26.4 (0.7) | **47.9 (2.5)** | **58.1 (1.4)** | **65 (2.2)** | **70.8 (2.6)** |
| | APTT-R | 0.84 – 1.16 | 1.05 (0.03) | **1.91 (0.10)** | **2.32 (0.06)** | **2.6 (0.09)** | **2.83 (0.11)** |
| **Dade® Actin® FSL** | APTT (s) | 25.0 – 32.0 | 27.1 (0.2) | **41.3 (1.3)** | **49.5 (3.3)** | **54.7 (3.4)** | **58.1 (2.8)** |
| | APTT-R | 1.00 – 1.28 | 1.08 (0.01) | **1.65 (0.06)** | **1.98 (0.13)** | **2.19 (0.15)** | **2.32 (0.13)** |
| **Dade® Thrombin** | FIB (g/L) | 1.70 – 4.10 | 2.54 (0.11) | 2.54 (0.13) | 2.64 (0.12) | 2.61 (0.09) | 2.54 (0.13) |
| **INNOVANCE® D-dimer** | DDi (mg/L) | < 0.5 | 0.26 (0.07) | 0.29 (0.07) | 0.29 (0.06) | 0.27 (0.01) | 0.24 (0.03) |

Data is presented as mean ± standard deviation. Data in bold indicate statistically significant differences (one-way ANOVA with post hoc Dunnett testing: $p < 0.0001$) compared to samples without asundexian (0 ng/mL). Abbreviations: APTT, activated partial thromboplastin time; APTT-R, activated partial thromboplastin time ratio; DDi, D-dimer; FIB, fibrinogen; INR, international normalised ratio; PT, prothrombin time. For each assay, five independent replicates of reconstituted standard human-derived plasma were spiked with asundexian or milvexian, respectively, were assayed ($n = 5$).

**Table S3.** Absolute mean values (standard deviation) of routine coagulation assay parameters in Control and Calibrator Material at select concentrations of asundexian

| | | | | **Asundexian** | | | | |
|---|---|---|---|---|---|---|---|---|
| **Assay** | **Control and Calibrator** | **Parameter** | **Reference Range** | **0 ng/mL** | **250 ng/mL** | **500 ng/mL** | **750 ng/mL** | **1000 ng/mL** |
| **Dade® Innovin®** | Ci-trol® 2 | PT (s) | 9.5 – 11.1 | 26.3 (0.5) | 26.2 (0.3) | 28.0 (0.9) | 26.8 (0.6) | 26.1 (0.5) |
| | | INR | 0.86 – 1.12 | 2.63 (0.05) | 2.63 (0.03) | 2.83 (0.09) | 2.69 (0.07) | 2.61 (0.05) |
| **Dade® Innovin®** | Ci-trol® 3 | PT (s) | 9.5 – 11.1 | 46.8 (1.1) | 45.9 (1.0) | 47.8 (1.0) | 46.6 (1.1) | 46.7 (1.2) |
| | | INR | 0.86 – 1.12 | 4.94 (0.1) | 4.84 (0.1) | 5.06 (0.1) | 4.92 (0.1) | 4.94 (0.1) |
| **Thromborel® S** | Ci-trol® 2 | PT (s) | 9.6 – 13.0 | 28.5 (0.4) | 28.9 (0.2) | 27.6 (0.9) | 27.1 (0.5) | 28.9 (0.6) |
| | | INR | 0.88 – 1.21 | 2.82 (0.04) | 2.90 (0.02) | 2.80 (0.10) | 2.85 (0.06) | 2.82 (0.07) |
| **Thromborel® S** | Ci-trol® 3 | PT (s) | 9.6 – 13.0 | 47.2 (0.2) | 48.0 (0.8) | 48.0 (0.8) | 48.6 (0.3) | 47.5 (0.7) |
| | | INR | 0.88 – 1.21 | 4.99 (0.02) | 5.08 (0.09) | 5.08 (0.10) | 5.16 (0.03) | 5.02 (0.08) |
| **Dade® Actin® FS** | Ci-trol® 2 | APTT (s) | 21.0 – 29.0 | 45.9 (0.7) | **51.8 (0.9)** | **70.0 (1.6)** | **86.0 (3.0)** | **91.6 (1.7)** |
| | | APTT-R | 0.84 – 1.16 | 1.83 (0.03) | **2.07 (0.03)** | **2.80 (0.06)** | **3.35 (0.12)** | **3.56 (0.06)** |
| **Dade® Actin® FS** | Ci-trol® 3 | APTT (s) | 21.0 – 29.0 | 63.7 (0.5) | **76.2 (3.5)** | **121. (6.7)** | **146.1 (5.3)** | **165.2 (2.3)** |
| | | APTT-R | 0.84 – 1.16 | 2.54 (0.02) | **3.04 (0.14)** | **4.85 (0.26)** | **5.85 (0.21)** | **6.60 (0.09)** |
| **Dade® Actin® FSL** | Ci-trol® 2 | APTT (s) | 25.0 – 32.0 | 44.4 (1.1) | **51.9 (0.5)** | **59.5 (0.3)** | **58.6 (6.5)** | **74.6 (3.6)** |
| | | APTT-R | 1.00 – 1.28 | 1.77 (0.04) | **2.07 (0.02)** | **2.38 (0.01)** | **2.34 (0.26)** | **2.98 (0.14)** |
| **Dade® Actin® FSL** | Ci-trol® 3 | APTT (s) | 25.0 – 32.0 | 68.1 (1.5) | **72.0 (1.9)** | **83.2 (1.1)** | **101.0 (3.5)** | **133.5 (4.4)** |
| | | APTT-R | 1.00 – 1.28 | 2.72 (0.06) | **2.88 (0.07)** | **3.32 (0.04)** | **4.04 (0.14)** | **5.34 (0.17)** |
| **Dade® Thrombin** | Control Plasma P | FIB (g/L) | 1.70 – 4.10 | 0.73 (0.04) | 0.71 (0.02) | 0.72 (0.03) | 0.66 (0.02) | 0.71 (0.04) |
| **INNOVANCE® D-dimer** | Control 2 | DDi (mg/L) | < 0.5 | 2.49 (0.04) | 2.62 (0.16) | 2.57 (0.15) | 2.5 (0.10) | 2.44 (0.05) |

Data is presented as mean ± standard deviation. Data in bold indicate statistically significant differences (one-way ANOVA with post hoc Dunnett testing: $p < 0.001$) compared to samples without asundexian (0 ng/mL). Abbreviations: APTT, activated partial thromboplastin time; APTT-R, activated partial thromboplastin time ratio; DDi, D-dimer; FIB, fibrinogen; INR, international normalised ratio; PT, prothrombin time. For each assay, three independent replicates of reconstituted standard human-derived plasma were spiked with asundexian or milvexian, respectively, were assayed ($n = 3$).

**Table S4.** Absolute mean values (standard deviation) of routine coagulation assay parameters in Control and Calibrator Material at select concentrations of milvexian

| | | | | **Milvexian** | | | | |
|---|---|---|---|---|---|---|---|---|
| **Assay** | | **Parameter** | **Reference Range** | **0 ng/mL** | **250 ng/mL** | **500 ng/mL** | **750 ng/mL** | **1000 ng/mL** |
| **Dade® Innovin®** | Ci-trol® 2 | PT (s) | 9.5 – 11.1 | 26.4 (1.4) | 26.6 (1.3) | 26.2 (1.2) | 25.9 (0.3) | 26.4 (1.1) |
| | | INR | 0.86 – 1.12 | 2.64 (0.15) | 2.67 (0.15) | 2.63 (0.13) | 2.60 (0.03) | 2.64 (0.12) |
| **Dade® Innovin®** | Ci-trol® 3 | PT (s) | 9.5 – 11.1 | 45.9 (1.2) | 45.6 (1.7) | 46.4 (1.8) | 46.6 (1.0) | 44.9 (0.7) |
| | | INR | 0.86 – 1.12 | 4.84 (0.14) | 4.81 (0.20) | 4.90 (0.21) | 4.92 (0.12) | 4.73 (0.08) |
| **Thromborel® S** | Ci-trol® 2 | PT (s) | 9.6 – 13.0 | 27.1 (0.4) | 28.8 (0.6) | 28.7 (0.3) | 28 (0.6) | 28.2 (0.6) |
| | | INR | 0.88 – 1.21 | 2.85 (0.05) | 2.80 (0.05) | 2.83 (0.02) | 2.86 (0.02) | 2.85 (0.07) |
| **Thromborel® S** | Ci-trol® 3 | PT (s) | 9.6 – 13.0 | 47.2 (0.2) | 48.0 (0.8) | 48.3 (0.9) | 48.6 (0.3) | 47.5 (0.7) |
| | | INR | 0.88 – 1.21 | 4.99 (0.02) | 5.08 (0.09) | 5.12 (0.10) | 5.16 (0.03) | 5.02 (0.08) |
| **Dade® Actin® FS** | Ci-trol® 2 | APTT (s) | 21.0 – 29.0 | 43.0 (0.5) | **51.8 (1.0)** | **69.0 (2.0)** | **89.0 (1.3)** | **104.4 (1.3)** |
| | | APTT-R | 0.84 – 1.16 | 1.72 (0.02) | **2.07 (0.04)** | **2.76 (0.08)** | **3.56 (0.05)** | **4.17 (0.05)** |
| **Dade® Actin® FS** | Ci-trol® 3 | APTT (s) | 21.0 – 29.0 | 68.2 (1.4) | **105.3 (9.0)** | **140.3 (7.6)** | **163.5 (2.6)** | **187.4 (1.3)** |
| | | APTT-R | 0.84 – 1.16 | 2.72 (0.05) | **3.96 (0.36)** | **5.61 (0.30)** | **6.54 (0.10)** | **7.49 (0.05)** |
| **Dade® Actin® FSL** | Ci-trol® 2 | APTT (s) | 25.0 – 32.0 | 43.4 (1.2) | **51.2 (0.4)** | **62.9 (0.4)** | **77.9 (2.2)** | **85.8 (1.1)** |
| | | APTT-R | 1.00 – 1.28 | 1.73 (0.05) | **2.04 (0.01)** | **2.51 (0.01)** | **3.11 (0.09)** | **3.43 (0.04)** |
| **Dade® Actin® FSL** | Ci-trol® 3 | APTT (s) | 25.0 – 32.0 | 65.8 (1.7) | **78.0 (3.5)** | **92.7 (0.7)** | **108.0 (1.8)** | **137.8 (7.4)** |
| | | APTT-R | 1.00 – 1.28 | 2.63 (0.06) | **3.12 (0.14)** | **3.71 (0.02)** | **4.32 (0.07)** | **5.51 (0.29)** |
| **Dade® Thrombin** | Control Plasma P | FIB (g/L) | 1.70 – 4.10 | 0.73 (0.04) | 0.71 (0.02) | 0.72 (0.03) | 0.66 (0.02) | 0.71 (0.04) |
| **INNOVANCE® D-dimer** | Control 2 | DDi (mg/L) | < 0.5 | 2.49 (0.04) | 2.62 (0.16) | 2.57 (0.15) | 2.50 (0.09) | 2.44 (0.05) |

Data is presented as mean ± standard deviation. Data in bold indicate statistically significant differences (one-way ANOVA with post hoc Dunnett testing: $p < 0.001$) compared to samples without asundexian (0 ng/mL). Abbreviations: APTT, activated partial thromboplastin time; APTT-R, activated partial thromboplastin time ratio; DDi, D-dimer; FIB, fibrinogen; INR, international normalised ratio; PT, prothrombin time. For each assay, three independent replicates of reconstituted standard human-derived plasma were spiked with asundexian or milvexian, respectively, were assayed ($n = 3$).

**Table S5.** Absolute mean values (standard deviation) of APTT-based assay parameters in human plasmas at different concentrations of asundexian

| | | | **Asundexian** | | | | |
|---|---|---|---|---|---|---|---|
| **Assay** | **Parameter** | **Reference Range** | **0 ng/mL** | **1.565 ng/mL** | **3.125 ng/mL** | **6.25 ng/mL** | **12.5 ng/mL** |
| **Dade® Actin® FS** | APTT (s) | 21.0 – 29.0 | 27.0 (3.6) | 27.2 (3.5) | 27.5 (3.4) | 28.3 (3.2) | 29.2 (3.0) |
| | APTT-R | 0.96 – 1.12 | 1.08 (0.15) | 1.09 (0.14) | 1.10 (0.14) | 1.13 (0.13) | 1.16 (0.12) |
| **Dade® Actin® FS** | FXI (%) | 67 - 127 | 87.5 (2.7) | 87.2 (2.7) | 86.9 (2.5) | 85.9 (3.0) | 85.5 (2.6) |

| **Assay** | **Parameter** | **Reference Range** | **25 ng/mL** | **50 ng/mL** | **100 ng/mL** | **200 ng/mL** | **300 ng/mL** |
|---|---|---|---|---|---|---|---|
| **Dade® Actin® FS** | APTT (s) | 21.0 – 29.0 | 31.0 (2.8) | 34.0 (2.5) | 39.4 (3.4) | 47.8 (3.0) | 50.3 (4.5) |
| | APTT-R | 0.96 – 1.12 | 1.24 (0.11) | 1.36 (0.10) | 1.57 (0.14) | 1.91 (0.12) | 2.01 (0.18) |
| **Dade® Actin® FS** | FXI (%) | 67 - 127 | 83.1 (3.0) | 79.4 (3.2) | 72.1 (3.1) | 61.8 (3.9) | 62.8 (12.) |

| **Assay** | **Parameter** | **Reference Range** | **400 ng/mL** | **500 ng/mL** | **600 ng/mL** | **700 ng/mL** | **800 ng/mL** |
|---|---|---|---|---|---|---|---|
| **Dade® Actin® FS** | APTT (s) | 21.0 – 29.0 | 58.5 (3.9) | 62.4 (4.2) | 65.8 (4.0) | 68.4 (4.4) | 71.0 (4.1) |
| | APTT-R | 0.96 – 1.12 | 2.34 (0.16) | 2.49 (0.17) | 2.63 (0.16) | 2.73 (0.18) | 2.84 (0.17) |
| **Dade® Actin® FS** | FXI (%) | 67 - 127 | 47.6 (5.5) | 43.0 (6.5) | 38.6 (7.4) | 35.3 (7.9) | 32.9 (7.7) |

| **Assay** | **Parameter** | **Reference Range** | **900 ng/mL** | **1000 ng/mL** | **2000 ng/mL** | **3000 ng/mL** | **4000 ng/mL** |
|---|---|---|---|---|---|---|---|
| **Dade® Actin® FS** | APTT (s) | 21.0 – 29.0 | 72.8 (4.6) | 75.1 (4.4) | 84.0 (5.3) | 91.6 (6.3) | 96.8 (5.6) |
| | APTT-R | 0.96 – 1.12 | 2.91 (0.19) | 3.00 (0.18) | 3.36 (0.21) | 3.66 (0.25) | 3.87 (0.23) |
| **Dade® Actin® FS** | FXI (%) | 67 - 127 | 30.7 (7.7) | 29.0 (7.4) | 22.9 (6.0) | 16.4 (3.4) | 14.1 (3.4) |

Data is presented as mean ± standard deviation. Abbreviations: APTT, activated partial thromboplastin time; APTT-R, activated partial thromboplastin time ratio; FXI, factor XI. For each assay, five independent replicates of five different reconstituted human-derived plasmas (Standard Human Plasma, Ci-trol® 1, Control Plasma N, BIOPHEN ™ Plasma Calibrator, and BIOPHEN ™ Normal Control Plasma) were spiked with asundexian or milvexian, respectively, were assayed (N = 5; *n* = 5).

**Table S6.** Absolute mean values (standard deviation) of APTT-based assay parameters in human plasmas at different concentrations of milvexian

| | | | Milvexian | | | | |
|---|---|---|---|---|---|---|---|
| **Assay** | **Parameter** | **Reference Range** | **0 ng/mL** | **1.565 ng/mL** | **3.125 ng/mL** | **6.25 ng/mL** | **12.5 ng/mL** |
| **Dade® Actin® FS** | APTT (s) | 21.0 – 29.0 | 26.9 (3.1) | 27.5 (3.0) | 27.9 (3.0) | 28.7 (3.0) | 29.7 (2.9) |
| | APTT-R | 0.96 – 1.12 | 1.07 (0.13) | 1.10 (0.12) | 1.11 (0.12) | 1.15 (0.21) | 1.18 (0.12) |
| **Dade® Actin® FS** | FXI (%) | 67 - 127 | 87.2 (6.3) | 87.2 (6.1) | 86.8 (6.0) | 85.9 (6.0) | 85.1 (5.8) |

| **Assay** | **Parameter** | **Reference Range** | **25 ng/mL** | **50 ng/mL** | **100 ng/mL** | **200 ng/mL** | **300 ng/mL** |
|---|---|---|---|---|---|---|---|
| **Dade® Actin® FS** | APTT (s) | 21.0 – 29.0 | 31.8 (2.8) | 34.8 (2.9) | 40.1 (3.1) | 49.8 (2.9) | 46.4 (6.4) |
| | APTT-R | 0.96 – 1.12 | 1.27 (0.12) | 1.39 (0.12) | 1.60 (0.13) | 1.99 (0.12) | 1.85 (0.26) |
| **Dade® Actin® FS** | FXI (%) | 67 - 127 | 82.6 (5.5) | 78.4 (5.2) | 70.1 (5.3) | 59.1 (5.9) | 55.7 (12.) |

| **Assay** | **Parameter** | **Reference Range** | **400 ng/mL** | **500 ng/mL** | **600 ng/mL** | **700 ng/mL** | **800 ng/mL** |
|---|---|---|---|---|---|---|---|
| **Dade® Actin® FS** | APTT (s) | 21.0 – 29.0 | 57.4 (4.2) | 61.3 (4.0) | 65.7 (3.5) | 68.8 (3.3) | 71.9 (3.3) |
| | APTT-R | 0.96 – 1.12 | 2.29 (0.17) | 2.45 (0.16) | 2.63 (0.14) | 2.75 (0.13) | 2.87 (0.14) |
| **Dade® Actin® FS** | FXI (%) | 67 - 127 | 44.5 (7.1) | 39.8 (7.5) | 35.7 (7.8) | 32.7 (8.0) | 30.4 (7.9) |

| **Assay** | **Parameter** | **Reference Range** | **900 ng/mL** | **1000 ng/mL** | **2000 ng/mL** | **3000 ng/mL** | **4000 ng/mL** |
|---|---|---|---|---|---|---|---|
| **Dade® Actin® FS** | APTT (s) | 21.0 – 29.0 | 74.3 (3.5) | 77.7 (3.5) | 87.7 (4.3) | 100. (6.5) | 108. (8.2) |
| | APTT-R | 0.96 – 1.12 | 2.97 (0.14) | 3.11 (0.14) | 3.50 (0.17) | 4.02 (0.26) | 4.35 (0.33) |
| **Dade® Actin® FS** | FXI (%) | 67 - 127 | 28.4 (7.8) | 26.7 (7.8) | 20.7 (6.5) | 15.6 (4.1) | 13.7 (3.8) |

Data is presented as mean ± standard deviation. Abbreviations: APTT, activated partial thromboplastin time; APTT-R, activated partial thromboplastin time ratio; FXI, factor XI. For each assay, five independent replicates of five different reconstituted human-derived plasmas (Standard Human Plasma, Ci-trol® 1, Control Plasma N, BIOPHEN ™ Plasma Calibrator, and BIOPHEN ™ Normal Control Plasma) were spiked with asundexian or milvexian, respectively, were assayed (N = 5; *n* = 5).

**Table S7.** Multi-dilutional analysis of factor XI activity in human plasmas at 1000 ng/mL of asundexian and milvexian

| | | | Asundexian (1000 ng/mL) | | | Milvexian (1000 ng/mL) | | |
|---|---|---|---|---|---|---|---|---|
| | | | Dilutions | | | Dilutions | | |
| **Assay** | **Parameter** | **Reference Range** | **1:1** | **1:2** | **1:4** | **1:1** | **1:2** | **1:4** |
| **Dade® Actin® FS** | FXI (%) | 67 - 127 | 29.0 (7.4) | 35.2 (6.1) | 44.9 (6.5) | 26.7 (7.8) | 34.2 (4.1) | 41.9 (4.5) |

Data is presented as mean ± standard deviation. Dilutions are presented as the volumetric ratio of asundexian- or milvexian-containing plasma and factor XI-deficient plasma assayed. Abbreviations: FXI, factor XI. For each assay, five independent replicates of five different reconstituted human-derived plasmas (Standard Human Plasma, Ci-trol® 1, Control Plasma N, BIOPHEN ™ Plasma Calibrator, and BIOPHEN ™ Normal Control Plasma) were spiked with asundexian or milvexian, respectively, were assayed (N = 5; *n* = 5).
